# “The landscape of love”: sex-specific habitat-use during the mating season in a solitary large carnivore

**DOI:** 10.1007/s10980-025-02250-6

**Published:** 2025-11-20

**Authors:** Alejandra Zarzo-Arias, Rick W. Heeres, Anne G. Hertel, Martin Leclerc, Shane Frank, Sam M. J. G. Steyaert, Jonas Kindberg, Jon E. Swenson, Vincenzo Penteriani, Fanie Pelletier, Andreas Zedrosser

**Affiliations:** 1 Department of Biology, Universidad Autónoma de Madrid, Madrid, Spain; 2 Department of Organisms and Systems Biology, Universidad de Oviedo, Oviedo, Asturias Spain; 3 Department of Natural Sciences and Environmental Health, University of South-Eastern Norway, Bø, Norway; 4 Department of Biology, Behavioural Ecology, Ludwig-Maximilian University of Munich, Planegg-Martinsried, Germany; 5 Département des Sciences Fondamentales & Centre d’étude de la Forêt, Université du Québec à Chicoutimi, Chicoutimi, QC Canada; 6 Mammals Research Section, Colorado Parks and Wildlife, Fort Collins, CO USA; 7 Terrestrial Ecology Group, Faculty of Biosciences and Aquaculture, Nord University, Steinkjer, Norway; 8 Norwegian Institute for Nature Research, Trondheim, Norway; 9 Department of Wildlife, Fish and Environmental Studies, Swedish University of Agricultural Sciences, Umeå, Sweden; 10 Faculty of Environmental Sciences and Natural Resource Management, Norwegian University of Life Sciences, Ås, Norway; 11 Department of Evolutionary Ecology, National Museum of Natural Sciences, Spanish National Research Council, Madrid, Spain; 12 Département de Biologie, Université de Sherbrooke, Sherbrooke, QC Canada; 13 Institute for Wildlife Biology and Game Management, University for Natural Resources and Life Sciences, Vienna, Austria

**Keywords:** Brown bear, *Ursus arctos*, Resource selection, Movement behavior, Social behavior, Reproduction

## Abstract

**Context:**

In mammals, reproductive strategies and movement behavior can differ between sexes, influenced by biological and environmental factors. Whereas males typically adopt a “roam-to-mate” strategy, increasing movement to locate females, females may also adjust their behavior to enhance mating opportunities. Habitat and human disturbance can further shape the spatial structure of mating encounters.

**Objectives:**

This study investigates sex-specific habitat use during mating in brown bears. We test (1) which habitats facilitate initial male–female encounters, and (2) how habitat use differs between solitary and consorting individuals, focusing on sex-based differences and responses to anthropogenic features.

**Methods:**

We used GPS data from 70 unique adult brown bears (44 females, 26 males) during the mating season in Sweden (2006–2016). We contrasted initial encounter areas of male–female pairs with surrounding available habitat to assess encounter site preferences, accounting for natural and anthropogenic landscape features. Additionally, we compared habitat use for each sex when solitary versus consorting.

**Results:**

Bears most often encountered the opposite sex in clearcuts and young forests. When consorting, males moved farther away from anthropogenic areas than when solitary and increased their use of clearcuts, whereas females reduced their use of young and old forests, in contrast to males. Both sexes approached roads more when consorting.

**Conclusion:**

This study revealed distinct sex-specific habitat preferences during brown bear consorting behavior, supporting the evidence of female “roam-to-mate” behavior by adjusting to males’ habitat use. Our findings emphasize the importance of managing anthropogenic landscapes for conservation efforts, especially if they disrupt mating behaviors.

**Supplementary Information:**

The online version contains supplementary material available at 10.1007/s10980-025-02250-6.

## Introduction

Females and males often exhibit different reproductive behaviors and strategies, which result in intersexual differences in the allocation of energy for reproduction (Andersson 1994; Clutton-Brock 1991). In polygamous mammals, males typically engage in energetically costly behaviors to compete for mating opportunities (Clutton-Brock 1991). In comparison, females invest more into their offspring (Gittleman and Thompson 1988) and influence reproductive outcomes through mate choice based on indicators of male quality (Isaac 2005; McPherson and Chenoweth 2012; Weckerly 1998). These mating behaviors and strategies are reflected in sex-specific movement behaviors (Dahle and Swenson 2003c). In addition, habitat configuration plays a key role in shaping movement behavior (Giuntini and Pedruzzi 2023; He et al. 2019; Webber et al. 2023), and thereby potentially affects mating opportunities (i.e., frequency and nature of social and reproductive encounters). Certain habitat types may increase the likelihood of meeting potential partners due to high visibility, olfaction benefits, accessibility, resource availability, concealment, or protection (Banks et al. 2007; Myhre et al. 2013). For example, open habitats could facilitate a safer environment due to increased perception (visibility or scent) of both potential partners and dangers (Camp et al. 2012; Fogarty et al. 2018). Alternatively, dense habitats might provide cover from competitors or predators while consorting (Embar et al. 2011).

Habitat use (Conde et al. 2010; Morris 1984; Oliveira et al. 2018) and home-range size (Lindstedt et al. 1986; Ofstad et al. 2016) commonly vary between the sexes, especially in sexually dimorphic species (Blanckenhorn 2005). Meeting locations should generally reflect the habitat preferences of the more stationary sex. Female mammals tend to move less and have smaller home ranges or territories compared to males (Dahle and Swenson 2003b, 2003c), which often adopt a “roam-to-mate” strategy to maximize encounters with receptive females during the breeding season. This behavior is especially common in polygynous and sexually dimorphic species, where reproductive success is closely tied to access to multiple mates (Emlen and Oring 1977). The impact of habitat on animal movement, social interactions, and mating strategies is well established (Banks et al. 2007; Giuntini and Pedruzzi 2023; Hirth 1977; Myhre et al. 2013). To our knowledge, few studies of wild, solitary mammals have investigated habitat use while consorting with partners (Banks et al. 2007; Fernández-Gil et al. 2006; Lodé, 2011). Comparing the habitat use of individuals when solitary vs when with a partner, as well as identifying sex-specific differences, will provide new insights about mammalian mating behaviors and strategies.

The human impact on the landscape and habitats is pervasive (e.g., Dri et al. 2024; Rillig et al. 2021; Torres et al. 2016) and profoundly affects wildlife movement behavior (Hertel et al. 2025; Tucker et al. 2018), with species and even individuals exhibiting varying degrees of behavioral plasticity in response (Júnior et al. 2022). For example, clearcutting a forested area may increase nesting opportunities or food availability for some species while resulting in the loss of critical habitat components for others (Michał and Rafał, 2014; Sullivan et al. 1999). Furthermore, anthropogenic presence and proximity can alter general movement capabilities and behaviors (e.g., mating behavior), while certain human-modified habitats and structures may offer novel opportunities or advantages. Consequently, accounting for the extent and nature of human influence is essential when studying animal movement and habitat use (Gomez et al. 2025).

The brown bear (*Ursus arctos*) is a solitary and sexually dimorphic species with a polygamous mating system and male–male competition (Dahle and Swenson 2003a; Steyaert et al. 2012; Swenson et al. 2007). The mating season lasts from around early May to mid-July (Heeres et al. 2024; Steyaert et al. 2012). Brown bears are generally considered nonterritorial (Bellemain et al. 2006; Craighead et al. 1995) and their home range size and movement patterns are related to the availability of resources, such as food and mates (Dahle and Swenson 2003b; McLoughlin et al. 2000). A key feature of brown bear spatial and reproductive behavior is female philopatry (Støen et al. 2005) and male dispersal to avoid inbreeding (Zedrosser et al. 2007). However, also females can exhibit “roam-to-mate” behavior during the mating season to ensure optimal male selection opportunities (Steyaert et al. 2014). In general, brown bears in Scandinavia prefer forested habitats characterized by rugged terrain and low human disturbance (Martin et al. 2010; Moe et al. 2007; Nellemann et al. 2007; Steyaert et al. 2013, 2016). Both sexes select for young forest types and avoid bogs and other water-related habitats (Berg 2009; Leclerc et al. 2016). In addition, clearcuts, human-modified habitat types, are selected at the population level (Leclerc et al. 2016) as they may provide food resources (Frank et al. 2015; Stenset et al. 2016). However, habitat use during the mating season may deviate from these general patterns. During this period, solitary adult females and males typically avoid human settlements, whereas subadults and females with dependent offspring occur closer to them (Elfström et al. 2014a, b; Elfström et al. 2014a, b; Steyaert et al. 2016). Given the reproductive significance of this period, better understanding spatial behavior during this critical phase in a bear’s life is essential.

The aim of this study was to assess habitat features where female and male bears encounter each other during the mating season, and to test the hypothesis that this consorting behavior is driven by habitat use and movement behavior of females rather than of males. We compared the habitat preferences of both sexes during consorting to assess whether females may also exhibit adaptive shifts indicative of a roam-to-mate strategy. We use movement data of 70 brown bears (44 females, 26 males) to evaluate the importance of habitat and landscape features on the mating behavior of both sexes during the mating season. First, we aimed to identify female–male encounters (i.e., female–male dyads meeting in time and space) during the mating season. We then contrasted first encounter locations with the surrounding habitat availability to test whether bears are more likely to meet in specific habitats. We predicted (*prediction 1*) that first encounter locations occurred in open habitats, presumably due to their improved visibility and detectability of partners. Second, we compared the sex-specific movement paths and habitat use of solitary vs. consorting individuals to evaluate which sex showed the most pronounced differences. We predicted (*prediction 2*) that males changed their habitat use patterns more than females, as females are the more stationary sex. Even though females might show “roam-to-mate” behavior, characterized by faster movement rates during the mating season (Steyaert et al. 2014), they show stable habitat use throughout the year (Berg 2009). Therefore, we expected that males would display a larger difference in habitat use as they switch from their “roam-to-mate” behavior to mating events in female-favored habitats.

## Methods

### Study area and population

The ~ 6000 km^2^ study area is located in Dalarna and Gävleborg counties in south-central Sweden (Martin et al. 2010). Main landcover types include intensively managed coniferous forest dominated by Scots pine (*Pinus sylvestris*) and Norway spruce (*Pica abies*), interspersed with bogs and lakes (Nellemann et al. 2007). The area is rugged, and elevation ranges from 93 m (a.s.l.) in the southeast to 734 m in the northwest. Human settlements are primarily found in the north and south, with few high-traffic roads (0.14 km/km^2^), but cabins and low-traffic paved roads occur throughout the study area (0.3/km^2^ and 0.7 km/km^2^, respectively; Martin et al. 2010). Human activity mainly occurs during summer and fall, including the hunting season and berry and mushroom picking (Ordiz et al. 2011).

The brown bear population in Sweden was estimated at ~ 3300 individuals in 2008 (2968–3667, 95% CI; Kindberg et al. 2011), but was reduced to ~ 2757 in 2017 (2636–2877, 95% CI; Bischof et al. 2020) due to an increase in harvest (Swenson et al. 2017). Bear density is ~ 30 individuals per 1000 km^2^ (Solberg et al. 2006). The mating season in the study area lasts from mid-May until mid-July (Heeres et al. 2024; Steyaert et al. 2012).

### Bear captures and monitoring

We captured brown bears from a helicopter (2006–2016) using a remote drug delivery system (Dan-Inject, Børkop, Denmark). See Graesli et al. (2025) for further details on capture and handling. We equipped bears with GPS collars (GPS Plus; Vectronic Aerospace, Berlin, Germany) programed to relocate the bear every hour. All captured bears were part of the Scandinavian Brown Bear Research Project (SBBRP), and all experiments, captures, and handling were performed in accordance with relevant guidelines and regulations and were approved by the appropriate authority and ethical committee (Naturvårdsverket and Djurförsöksetiska nämnden i Uppsala, Sweden). In total, we compiled data on 143 unique collared bears (79 females and 64 males) between 2006 and 2016 (about 27 females and 13 males per year).

### Defining social encounters

To define an encounter between a male and a female (i.e., both individuals at the same location at the same time within set spatiotemporal thresholds), we explored patterns in the pairwise distance between all bear GPS relocations at a given time (rounded to the nearest hour) during the mating season (when we assume that bears are mostly focused on mate-seeking behaviors). We first assessed the number of encounters occurring at different spatial distances between bears. We observed a steep drop in the number of encounters with increasing distance, reaching a local minimum at 37 m (Fig. S1A), indicating that below this distance, males and females would come into close enough proximity to potentially mate. Considering GPS accuracy (± 15 m; Moe et al. 2007), we set the encounter distance threshold at 67 m (37 m plus two times the GPS error). Additionally, given that the GPS fix rate success was high (~ 95% meaning that we missed about 5% of fixes throughout the period an individual was followed; Leclerc et al. 2016), we imposed a temporal threshold to distinguish between continuous and intermittent encounters. The frequency of interactions dropped sharply as the time between events increased, with interaction frequency stabilizing around 3 h (Fig. S1B). Thus, we defined the temporal threshold, marking the end of an encounter as 3 consecutive hours without proximity. Consequently, an encounter event was defined as a period during which a female and a male were within 67 m for at least 3 consecutive hours. This ensured that we only classified sustained proximity as an encounter, excluding brief, incidental overlaps in the home range. According to population estimates (Solberg et al. 2006), tracked individuals represented approximately 50–70% of the local adult population. Although it remains possible that consorting events occurred between a collared and an uncollared individual, we are confident that most solitary paths reflected true solitary behavior (see Discussion for further argumentation).

### Environmental layers

To describe bear habitat use when alone or consorting during the mating season, we used the following landscape components important for bear ecology (Ordiz et al. 2011; Steyaert et al. 2013, 2016; Van de Walle et al. 2019; Zarzo-Arias et al. 2019): distances (m) to the (1) nearest major road (accessible for motorized vehicles and high traffic volume), (2) minor road (accessible for motorized vehicles and low traffic volume), (3) human settlements, and (4) single-standing buildings outside villages or human settlements; (5) the five main habitat types in the study area: young (tree height < 7 m, > 7 years old, ~ 13% of the study area), mid-age forest (tree height 7–15 m, ~ 31%) and old forest (tree height > 15 m, ~ 21%), bogs (wet peat ground with low productivity, ~ 10%), and clear-cuts (open habitats with no or recent tree plantations, ~ 12%); (6) elevation (DEM), and (*7*) terrain ruggedness. The land cover type raster (25 × 25 m^2^) came from an annually updated CORINE map obtained from the Swedish Forest Agency (Svensk MarktäckeData). The digital topographical map, digital elevation model, and human-related layers were obtained from the National Land Survey Sweden (www.lantmateriet.se, License No. I 2012/901). We used QGIS 2.18 and R 4.3.2. for all spatial processing (QGIS Development Team 2020; R Development Core Team 2023).

### First encounter and random locations

**Fig. 1:**
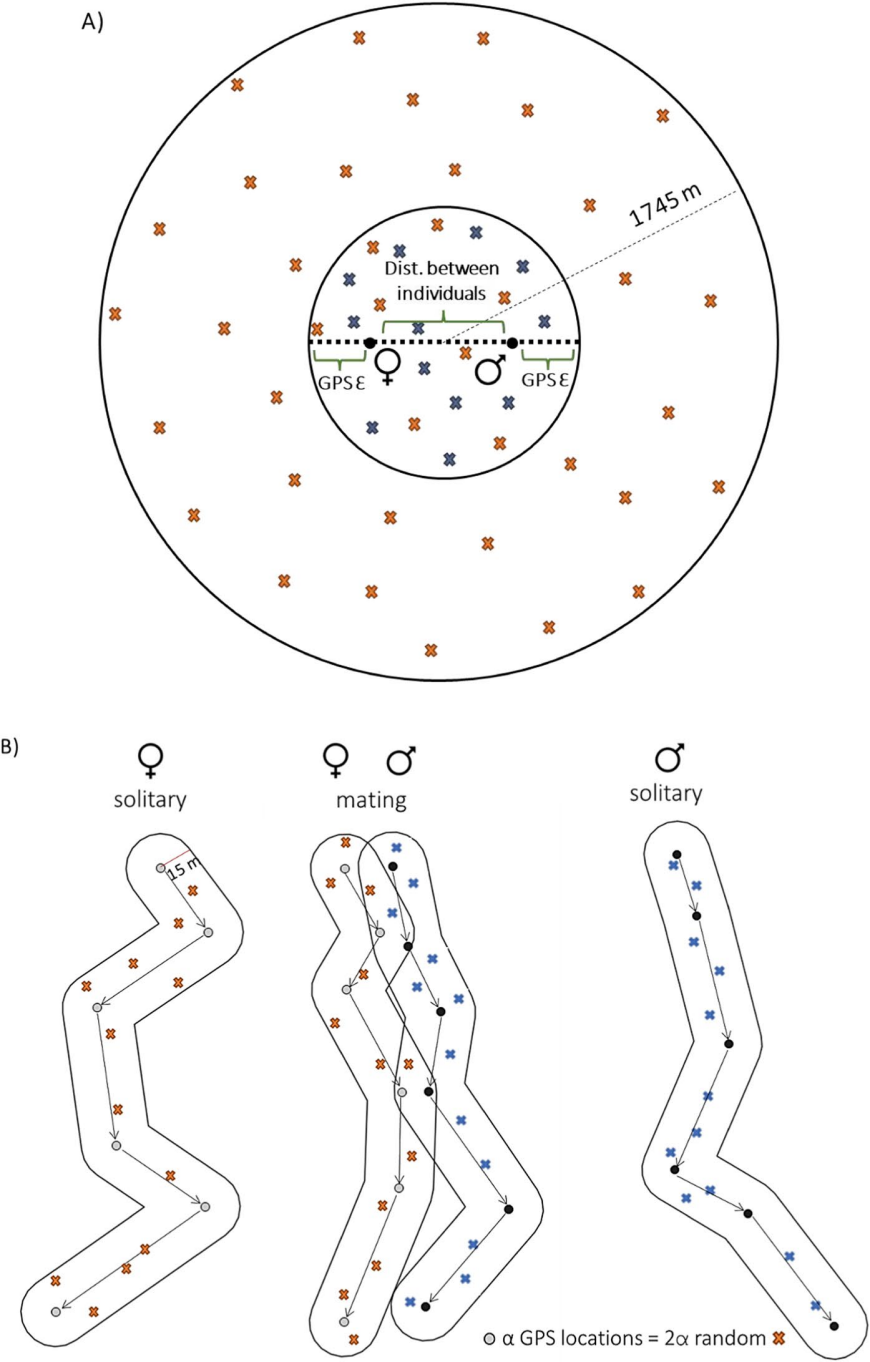
Representation of the method used for selecting random points for the analysis of habitat use by adult male and female brown bears during encounters in the mating season. **A** First encounter location with random points representing used (blue: 1 random point per 10 m^2^) and available (orange: 50 random points) habitats (inside a 1745-m buffer: average 3-h displacement of solitary adult bears during the mating season); **B** solitary and consorting paths with GPS relocations and random points sampling the used habitat of females (orange) and males (blue) at twice the number of GPS locations for each path

The first encounter location was defined as the center between the initial GPS coordinates of an adult male and female interacting during the mating season (Fig. 1A). The same couple can have more than one first encounter, if they are separated in time in the same or different years. To describe the habitat at the first encounter location, we created a buffer with a diameter of the distance between the two bears plus two times the GPS collar error (15 m). We generated one random location per 10 m^2^ surface area of an encounter buffer to describe the encounter location. Then, we sampled available habitat within a buffer with a radius of 1745 m centered around the encounter location to describe habitat availability. The buffer size was based on the average 3-h displacement of solitary adult bears during the mating season calculated as the Euclidean distance between a focal location and the location 3 h later using active positions only (hourly displacement > 20 m). We calculated displacement separately for females (1501 m) and males (1990 m) and took the average displacement of both as buffer radius (1745 m). We used 50 random locations, as they represented the local available habitat well (i.e., additional random points did not alter the proportional share of habitat types by > 1%; Serrouya et al. 2011; Fig. S2).

### Solitary and consorting paths

A consorting path was defined as the path of a male and a female within 67 m (i.e., an encounter) for at least three hours (i.e., three consecutive locations; Fig. S1). For every consorting path, we randomly selected a path from the same male and female when they were presumably solitary (i.e., > 1 km from all other GPS-collared bears). The selected solitary paths had the same length (e.g., equal number of GPS locations) as the consorting paths and were during the same mating season. For both the consorting and solitary paths, we created a 15-m buffer around each individual path (i.e., to account for location error). Within this buffer we created twice as many random locations as the number of recorded GPS locations (Fig. 1B).

### Statistical analyses

#### Initial encounters

We used mixed-effects logistic regression (GLMM) to fit a resource selection function (RSF; Johnson et al. 2006) using the *lme4* package (Bates et al. 2015), with ‘used’ random points (blue; coded 1) inside first encounter areas versus ‘available’ (1745 m radius buffer) random points (orange; coded 0) as the response variable (Fig. 1A). We defined a model combining distances to nearest human infrastructure, habitat, and terrain variables (variance inflation factors, VIF < 3; Akinwande et al. 2015). We considered all the habitat variables as fixed effects and scaled all continuous variables (mean = 0, variance = 1). From the habitat classes, we chose mid-aged forest as the reference category for contrast (~ intercept), because bears exhibited a selection ratio close to 1 for mid-aged forest (Table S1). We included *encounter ID* and *individual ID* as random intercepts due to repeated individual observations. *Encounter ID* (structured as *female ID* + *male ID* + *encounter number of the same couple*) is a unique tag for all locations inside each first encounter area (Fig. 1A). The *individual ID* was included to account for repeated observation of the same individual, as we might have several encounters from the same ID during a mating season or between years.

#### Habitat use during solitary vs consorting

To evaluate whether sex-specific changes in habitat use occur during consorting, we used mixed-effects logistic regression models utilizing the random locations for the solitary (0) and consorting (1) paths of either sex (Fig. 1B). We used these models to contrast solitary and consorting paths for each sex and evaluate if there were changes in habitat use. We considered all the landscape variables as fixed effects and we scaled continuous variables (mean = 0, variance = 1). We included sex as a factor interacting with all fixed effects to explore sex-differences in habitat use at the population level. Similarly to the initial encounter location analysis, mid-aged forest was selected as the reference category (Table S1). We included the scaled length of each path, measured as the number of GPS locations in each track (identical for the consorting and solitary paths within each encounter) as a predictor to account for variation in path size across encounters. *Path ID* and *individual ID* were included as random intercepts. *Path ID* refers to the consorting path of the couple, and the corresponding solitary paths for both the male and the female (structured as *female ID* + *male ID* + *path number* + *year*). The *individual ID* was used to consider repeated observations of the same individual, as we might have several paths from the same bear in a season or between years.

## Results

### Bear encounters

In total, we identified 427 encounters between 26 GPS-collared unique adult males (≥ 3 years old) and 44 GPS-collared unique solitary females (≥ 3 years old, not accompanied by dependent offspring of any age in the month of June). The number of collared individuals varied by year, because they may keep the collar (average for females = 3.9 years and males = 2.3 years), lose it, or die. For our study period, there was an annual mean of 27.4 collared females and 13.2 males, ranging from a minimum of 13 and 1 (on the first year of tracking), to a maximum of 34 and 22, respectively. The encounters lasted on average 20 h (median = 4.5 h; Fig. S3A), with the shortest encounters being less than 1 h (representing 29% of all events) and the longest 340 h (14 days, one event). Males consorted with 2.2 females per mating season on average (Figure S3B), and females with 1.6 males (range 1–5 different partners). For further details on male–female encounters and summary statistics of the encounter dataset, see Supplementary Figure S3.

### Where do bears consort in the landscape?

**Fig. 2:**
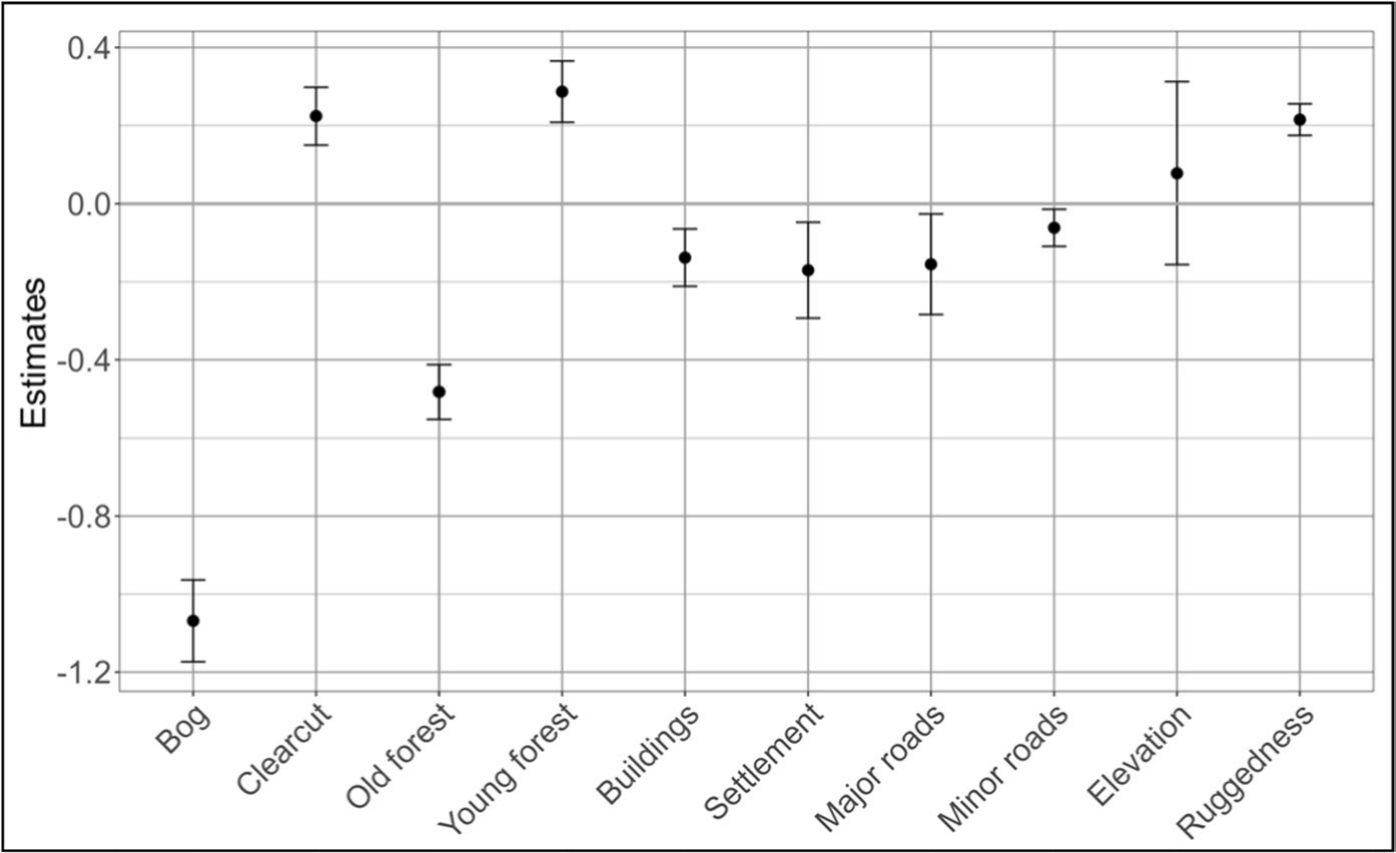
Model coefficients and 95% confidence intervals of the variables included in the model built to evaluate first encounter locations defining habitat compared to available habitat (1745-m radius buffer) during the mating season in south-central Sweden (2006–2016). Mid-age forest represents the category of reference (Bog, Clearcut, Old forest, and Young forest). “Distance to” coefficients (human-related variables) were reversed for easier interpretation, so positive values indicate selection (i.e., approaching) and negative values indicate avoidance (i.e., moving away). A significant effect is indicated when the CI does not cross the zero line, suggesting use (above zero) or avoidance (below zero)

In the model we compared the first encounter area and the available area surrounding it (1745-m buffer). The number of sample points ranged from 7 to 73, with a mean of 29 points. Pairs more strongly selected for clearcuts and young forests for their first encounters than mid-aged forests, and for higher altitudes and more rugged areas compared to available areas (Fig. 2; Table S2). Bears avoided bogs and old forests more strongly than mid-aged forest for their first encounters and they also avoided roads and human settlements (Fig. 2; Table S2).

### Sex-specific habitat use during consorting

**Fig. 3:**
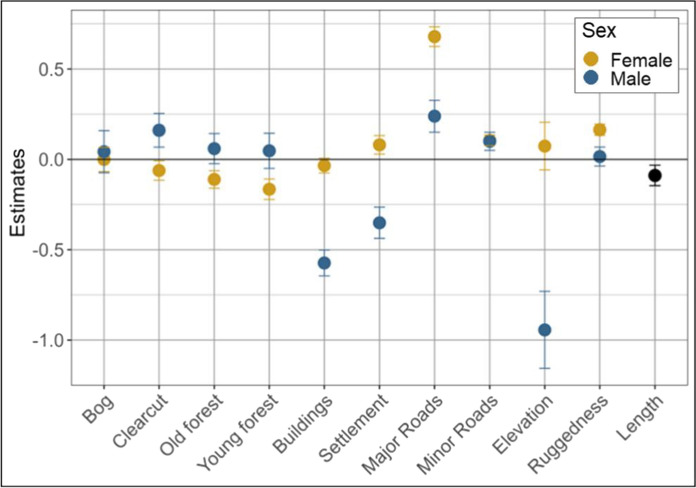
Model coefficients and 95% confidence intervals of the variables used in the model built to decipher solitary (coded 0) vs consorting (coded 1) paths for female and male brown bears in south-central Sweden (2006–2016). Mid-age forest represents the category of reference (Bog, Clearcut, Old forest, and Young forest). “Distance to” coefficients (human-related variables) were reversed for easier interpretation, so positive values indicate selection (i.e., approaching) and negative values indicate avoidance (i.e., moving away). A significant effect is indicated when the CI does not cross the zero line, suggesting use (above zero) or avoidance (below zero)

We identified 242 consorting paths from 24 unique males and 37 unique females, in 81 unique pairs. The consorting and solitary paths for each individual of the couple had the same number of relocations, ranging from 3 to 336 relocations, with a mean of 64. We found that both sexes exhibited different habitat use while consorting compared to being solitary, but these differences varied by sex (Fig. 3; Table S3). During consorting, both sexes moved closer to roads, particularly major roads. On the other hand, males moved away from settlements and buildings while consorting and to lower altitudes, whereas females moved slightly closer to settlements and selected for higher terrain ruggedness. Regarding habitat types, males marginally increased their use of clearcuts and old and young forest compared to when solitary, whereas females exhibited the opposite shifts.

## Discussion

By using a long-term movement dataset, we investigated patterns of female–male bear encounters during the mating season. We characterized which habitats and landscape characteristics were used for initiating consorting events (first encounters) by brown bears, and whether consorting paths more closely reflected the habitat use of solitary females or males. The extensive movement and environmental data allowed us to gain new insights into the mating behavior of a polygamous, solitary, and sexually dimorphic large carnivore. Our results, as expected (*prediction 1*), showed that consorting events were more likely initiated in clearcuts and young forests, whereas bogs and older forests were avoided. Also, we found that the first encounters occurred relatively far from human infrastructure. Contrary to our expectations (*prediction 2*), we found that both sexes showed different habitat selection while consorting compared to being solitary during the mating season. Males changed their consorting habitat selection especially in relation to anthropogenic factors, as they avoided buildings and settlements. While consorting, female brown bears decreased their selection of clearcuts, young, and old forests compared to when solitary, despite these habitats typically offering high seasonal food resource availability (Michał and Rafał, 2014; Twynham et al. 2021). Similar patterns were found for females and males regarding roads, as both moved closer to roads, especially major roads, when consorting. These differences in habitat use may be attributed to the distinct tactics employed by each sex during the mating season.

Clearcuts represent more open and likely safer habitats for bears, where it can be easier to detect (visually and by olfaction) conspecifics or potential threats (Camp et al. 2012; Myhre et al. 2013) allow individuals to focus on potential partners (Steyaert et al. 2013). This pattern also reflects the general use of clearcuts by female bears throughout their active period (for foraging, resting, traveling) in the study area (Berg 2009; Frank et al. 2015). Bear couples did not use bogs and old forests as first meeting locations, consistent with previous findings (Hertel et al. 2019; Leclerc et al. 2016). Bogs can significantly limit movement capabilities, whereas old forest patches (scarce in Sweden) might be used primarily during the hyperphagia season (i.e., period when bears solely focus on food resources after the mating season), as they normally harbor several berry species in the undergrowth layer (Hertel et al. 2016; Stenset et al. 2016). In highly managed forests, such as the Swedish boreal forest, clearcuts, young, and mid-age forest types are widespread, with only small, scattered patches of old forest remaining. It would therefore be valuable to investigate where bears interact in less intensively managed forest systems, particularly in areas where open or anthropogenically influenced habitats such as clearcuts are less prevalent than in our study area.

Generally, females focus on food and males on mates as critical resources during the mating season in polygamous species (Andersson 1994). In our study site, the main food resources available during this time of the year (May–July) are neonate ungulates (e.g., moose (*Alces alces*); Rauset et al. 2012; Twynham et al. 2021) and ants (*Formicidae* spp.; Stenset et al. 2016; Swenson et al. 1999), as berries only ripen after the mating season (Hertel et al. 2016). Moose often give birth  in concealed areas (e.g., high shrub and tree cover) such as young forest types, and away from anthropogenic areas, where they remain for the first months after birth (Bjørneraas et al. 2011; Melin et al. 2019). As predators on calves, brown bears select for similar habitats as moose during the calving season (Twynham et al. 2021). Ants are also a potential food source during the study period and are often available on clearcuts (Frank et al. 2015). Therefore, our study suggests that the habitats selected for initial meeting sites are used by females as they provide seasonal food resources and that males likely find potential partners in these areas.

The path analysis revealed an increased use of clearcuts by consorting males, likely because such habitats, along with areas near roads (Brown et al. 2024), may facilitate efficient movement and improve opportunities to initiate consorting with females. the use of clearcuts could also reflect males exploiting them for foraging, particularly for ants (Swenson et al. 1999). Even if males are expected to prioritize finding potential mating partners, a relatively abundant food resources in open areas may still be exploited. The use of roads as travel corridors by both sexes when solitary (Hill et al. 2021) may also enhance mating success, since these structures can benefit easier displacements through the landscape (Hill et al. 2021). These behaviors occur within a broader context of human disturbance, where animals generally avoid areas or at least alter their movement behavior, near anthropogenic activity (Hertel et al. 2025; Tucker et al. 2018). Among brown bears, males in particular show a clear avoidance of human presence (Ordiz et al. 2011; Van de Walle et al. 2019), whereas females with offspring often use these areas to avoid infanticidal males (Steyaert et al. 2016). Solitary males approaching human settlements and buildings likely prioritize mating opportunities rather than minimizing potential human disturbances, whereas during consorting, shared movements bring females closer to roads than they usually approach when alone.

Changes in habitat configuration and population dynamics can lead to adaptations by both sexes (He et al. 2019; Kvarnemo and Ahnesjö, 1996). For instance, shifts in adult sex ratio may cause the more sedentary sex to alter movements and habitat use (Eberhart-Phillips et al. 2017; Székely et al. 2014; Webber and Vander Wal 2018). The Swedish bear population is currently estimated to have an adult sex ratio of 41% males (range 39–44%) and 59% females (range 56–61%; Bischof et al. 2019), likely due to male-biased harvest (Bischof et al. 2008; Leclerc et al. 2019; Milner et al. 2007). Earlier research has suggested that females use a “roam-to-mate” behavior (Dahle and Swenson 2003c; Steyaert et al. 2014), which might be related to their release from maternal care, leading to increased movement and larger home ranges, but change in habitat use has not been investigated. According to our GPS data, 90% of females consorted with 1 or 2 different collared males during a specific mating season, whereas males met with 1 to 5 different collared females, showing a greater variance (Fig. S3). If densities are low, mating with two males might already ensure benefits such as a reduced chance for infanticide to occur (Penteriani et al., 2024; Steyaert et al. 2016) and might not force females to find more mates. However, we found contrasting habitat use patterns between sexes. We hypothesize that this behavior is due to females adjusting to males’ preferences and thus, also showing a “roam-to-mate” behavior, reflecting mutual behavioral adaptation and compromise.

Although our findings provide valuable insights into sex-specific habitat use during the mating season, several limitations should be acknowledged. First, due to logistical constraints, we could not monitor all adult individuals within the study area. Over the study period (2006–2016), 143 adult brown bears (79 females, 64 males) were GPS-collared (about 27 females and 13 males per year), which, according to population estimates (Solberg et al. 2006), represents approximately 50–70% of the local adult population. Although it remains possible that collared individuals consorted with uncollared individuals, we are confident that most solitary paths reflect true solitary behavior. This is further supported by our focus on consorting-related interactions, which we defined as common paths lasting at least 3 h—excluding 30% of encounters that were shorter and less likely to represent a mating event, because couples commonly traveled together while the male monitors the female’s reproductive status (Steyaert et al. 2012). Even if a collared bear encountered an uncollared individual of the opposite sex, it is unlikely that the solitary path would overlap substantially with another consorting event for most of its duration. This is a general caveat in studies involving social interactions, and we believe it does not significantly affect our main conclusions.

This study provides a sex-specific movement-based analysis of mating behavior in a wide-ranging, polygamous mammal. By linking fine-scale spatial data with behavioral ecology, our findings enhance understanding of how landscape and anthropogenic features influence reproductive interactions (He et al. 2019). These insights are crucial not only for advancing theoretical models of mating tactics and habitat use, but also for informing conservation strategies in human-dominated environments where movement and social behaviors may be disrupted. Overall, our findings underscored the importance of anthropogenically impacted habitats, such as clearcuts and areas near roads, for brown bears during mating, particularly in regions where human land use and activity increasingly overlap with bear habitats. This improved understanding of habitat use provides new insights into the conservation of brown bears in regions like Europe, where considering human infrastructure, forest management, and human-wildlife interactions is essential to sustain carnivore populations. Conservation strategies should potentially incorporate information regarding sex-based habitat preferences during mating to sustain important habitats for reproductive purposes (Conde et al. 2010; van Toor et al. 2011). This may involve protecting and managing habitats preferentially used when mating, for example, ensuring that clearcuts and young forests remain accessible and relatively undisturbed during the mating season. These targeted approaches differ from general habitat protection by recognizing and maintaining the distinct spatial needs of males and females during critical reproductive periods, which could improve mating success and population viability. Such considerations might be especially important for species that cannot cope with rapid changes, have low population densities and/or large home ranges, and slow life-history traits.

Therefore, it would be interesting to compare the habitats used for initial meeting locations and consorting among populations of the same species. Further research could explore how these shifts in habitat choice affect the reproductive success and overall fitness of mating pairs, as well as the potential influence of skewed sex ratios on mating strategies, offering additional valuable insights for conservation efforts (Banks et al. 2007; Giuntini and Pedruzzi 2023). To further unravel the potential use of “roam-to-mate” behavior by females in the Scandinavian brown bear population, a closer look at movement patterns prior to consorting encounters might shed light on this specific topic. Additionally, exploring the influence of expanding road networks on social interactions may reveal conflicting effects, necessitating careful consideration in their design and management to mitigate potential negative consequences on bear populations and their habitats. Furthermore, individual behavioral variation can strongly influence ecological processes and population-level patterns, particularly in species with complex social and reproductive strategies. Recognizing and incorporating this variation into future analyses could help identify alternative mating tactics, habitat-use flexibility, and context-dependent trade-offs that may be masked by population-level averages, thereby improving the predictive power of conservation models. Importantly, these efforts should be embedded within a broader ecological framework, ensuring that management actions benefiting bears do not inadvertently harm other species or reduce biodiversity. Conservation planning should balance these seasonal advantages with year-round habitat requirements, and with the broader ecological consequences for forest-specialist species and overall biodiversity.

## Supplementary Information

Below is the link to the electronic supplementary material.

Supplementary file1 (DOCX 607 kb)

## Data Availability

The data and code that support the findings of this study are available on GitHub/alezarzo.
