# Supplementary material for: “The landscape of love”: sex-specific habitat-use during the mating season in a solitary large carnivore
Source: Landsc Ecol. 2025 Nov 20;40(12):222. doi: 10.1007/s10980-025-02250-6 (PMC12642813; doi:10.1007/s10980-025-02250-6)
Supplement: Supplementary file 1 — Supplementary file1 (DOCX 607 kb) [file 10980_2025_2250_MOESM1_ESM.docx]

**Supplementary material**

**Figure S1.** Graphs illustrating our definition of a mating encounter between male and female brown bear in the mating season in Sweden. (A) Number of encounters considering different distances between the bears regarding a 3-hour interval between different encounters and (B) number of encounters considering different hours interval between two different encounters. Increasing the threshold to 100 m in Fig. S1A results in more encounters, as bears previously 50–90 m apart would now be included. Conversely, with a 20 m threshold, two bears 10 m apart at time *t*, then 40 m at *t+1*, and back to 10 m at *t+2* would count as two encounters, though it's likely a single prolonged one. This explains why encounters count rise as distance decreases in panel A. To avoid splitting continuous interactions into multiple encounters, we select the distance that minimizes total encounters and also apply a time gap criterion (panel B).


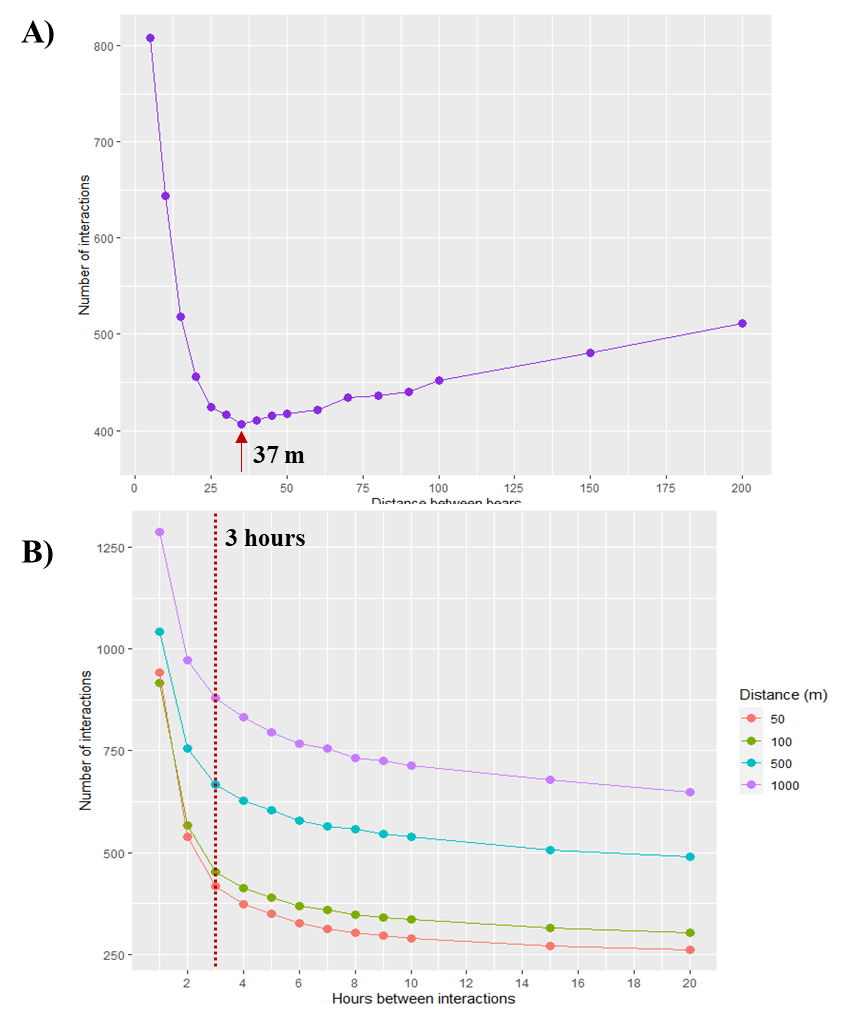


**Figure S2.** Percentage of habitat change composition in increasing number of random points for the 1745 meter radius buffer created to test habitat selection for the first mating encounter location between a female and a male brown bear in Sweden (Serrouya et al., 2011).


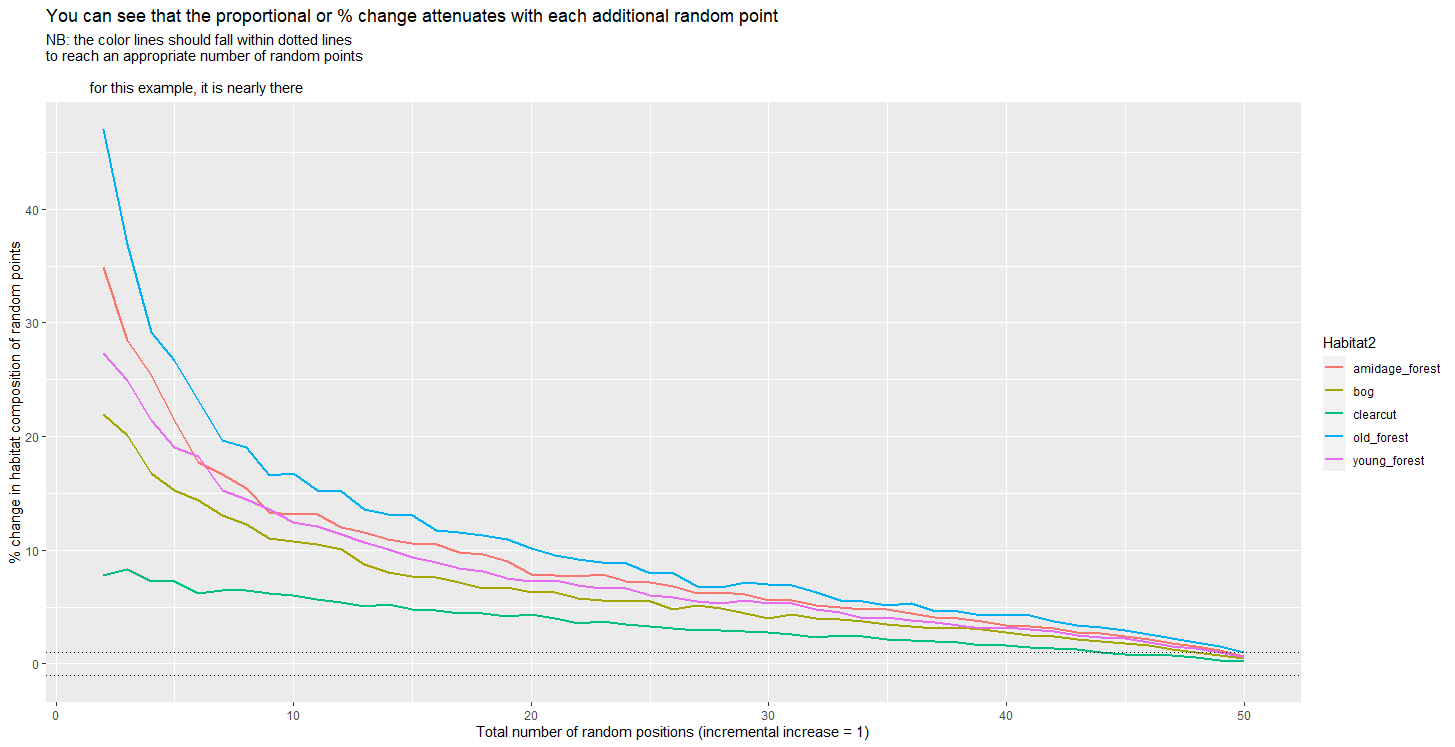

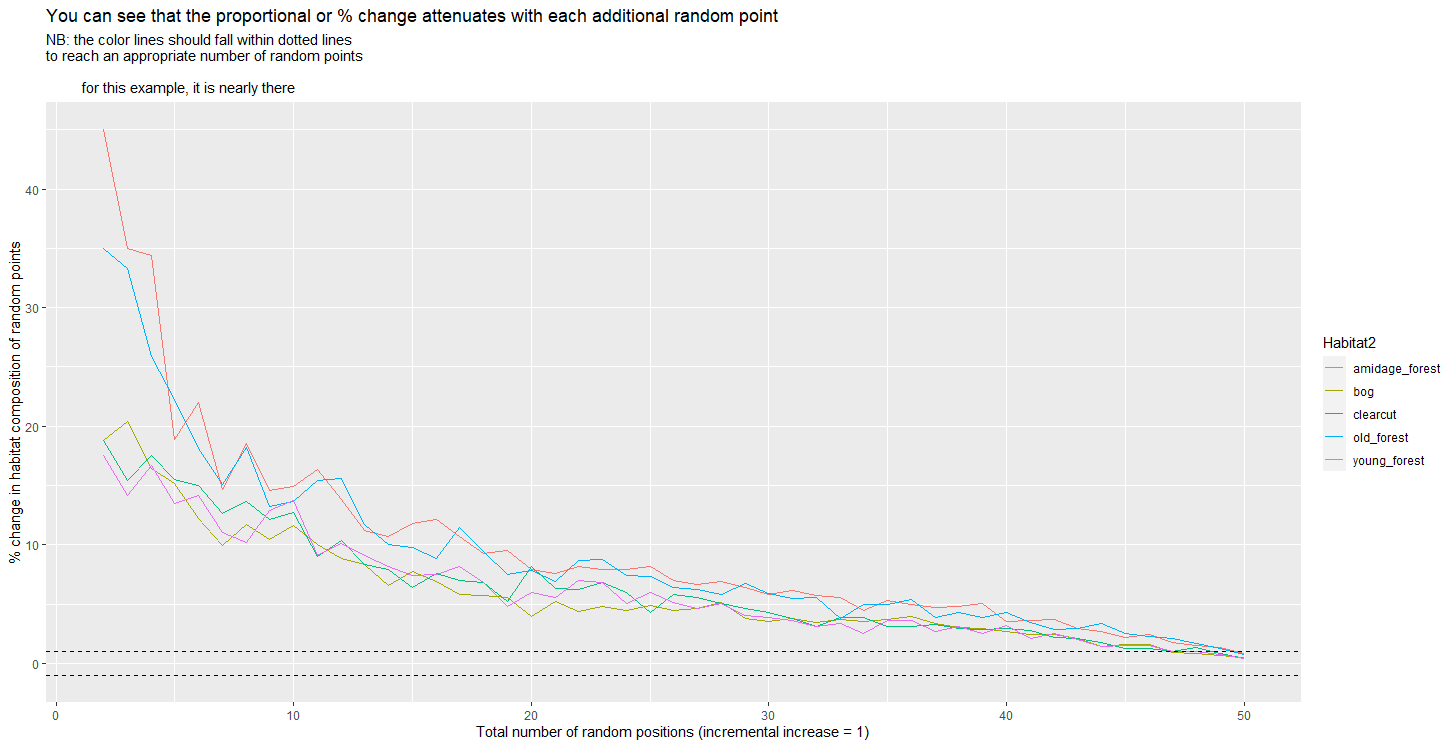

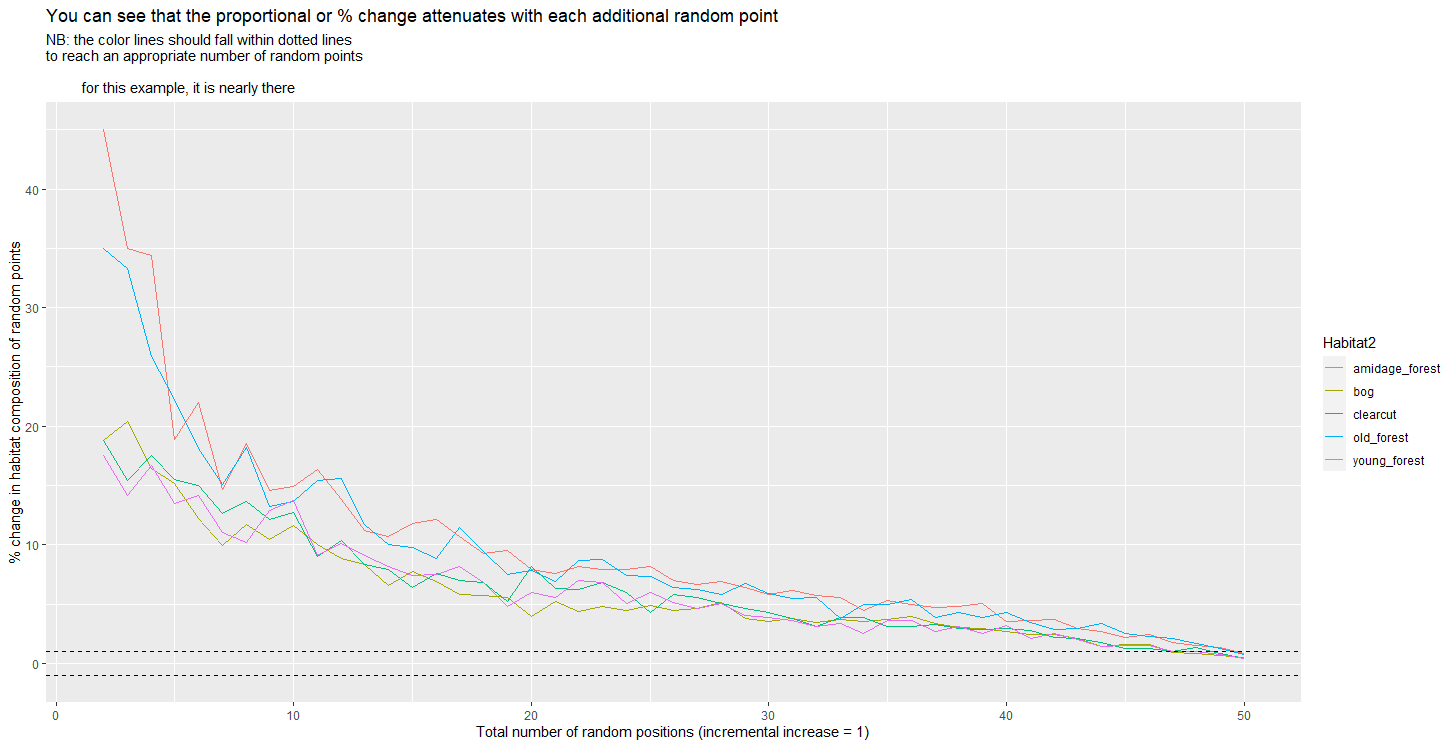


**Figure S3.** Descriptives of adult female and male brown bear encounters in Sweden: (A) histogram of encounters duration (in hours); (B) number of bears that had one or more different couples in the same year; and (C) number of encounters per sex and age (columns) corrected (i.e., divided) by the number of collared individuals of each age (lines).

**A)**

**
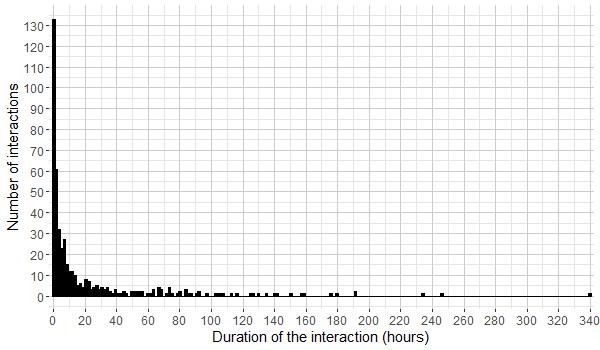
**

**B)**


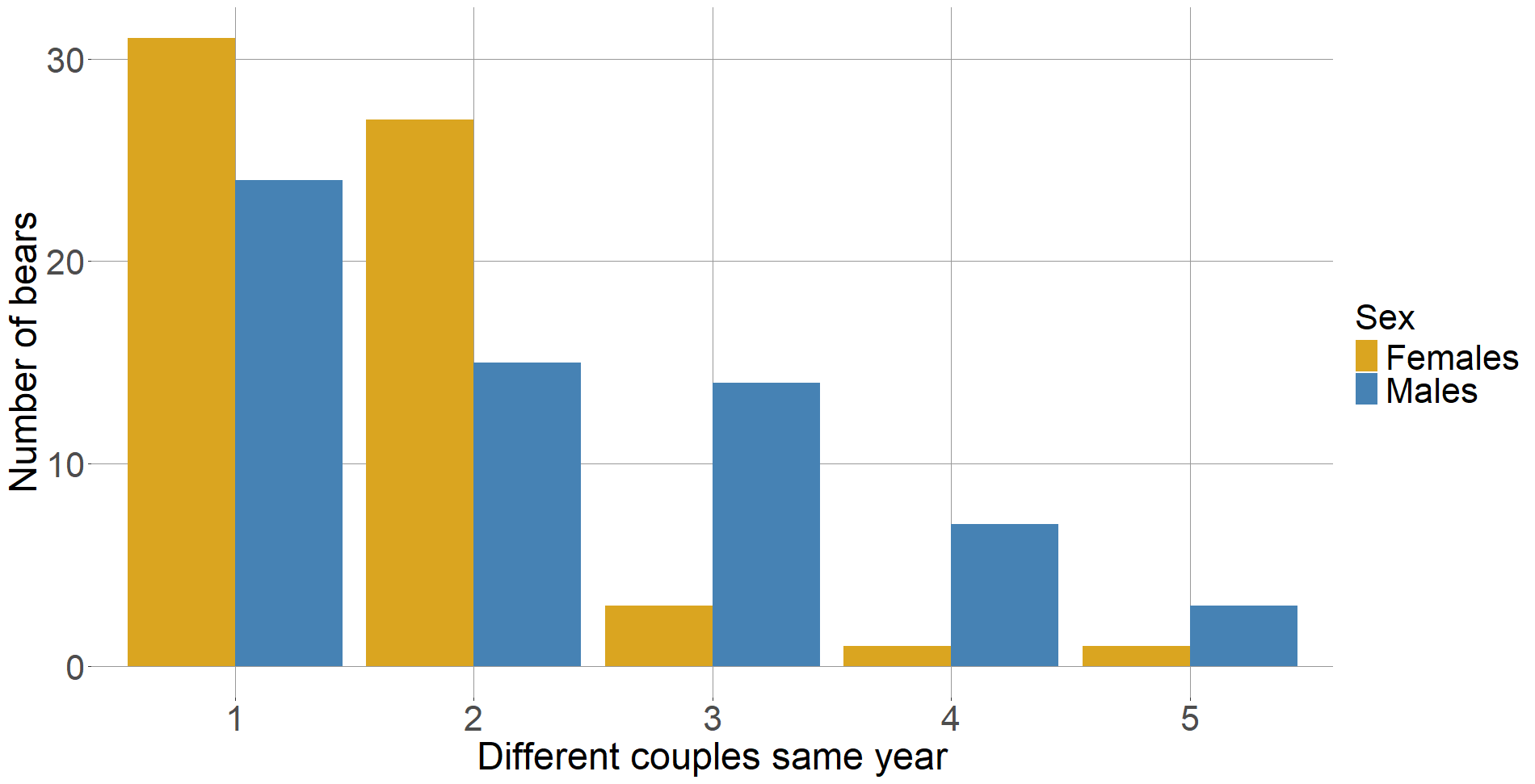


**C)**


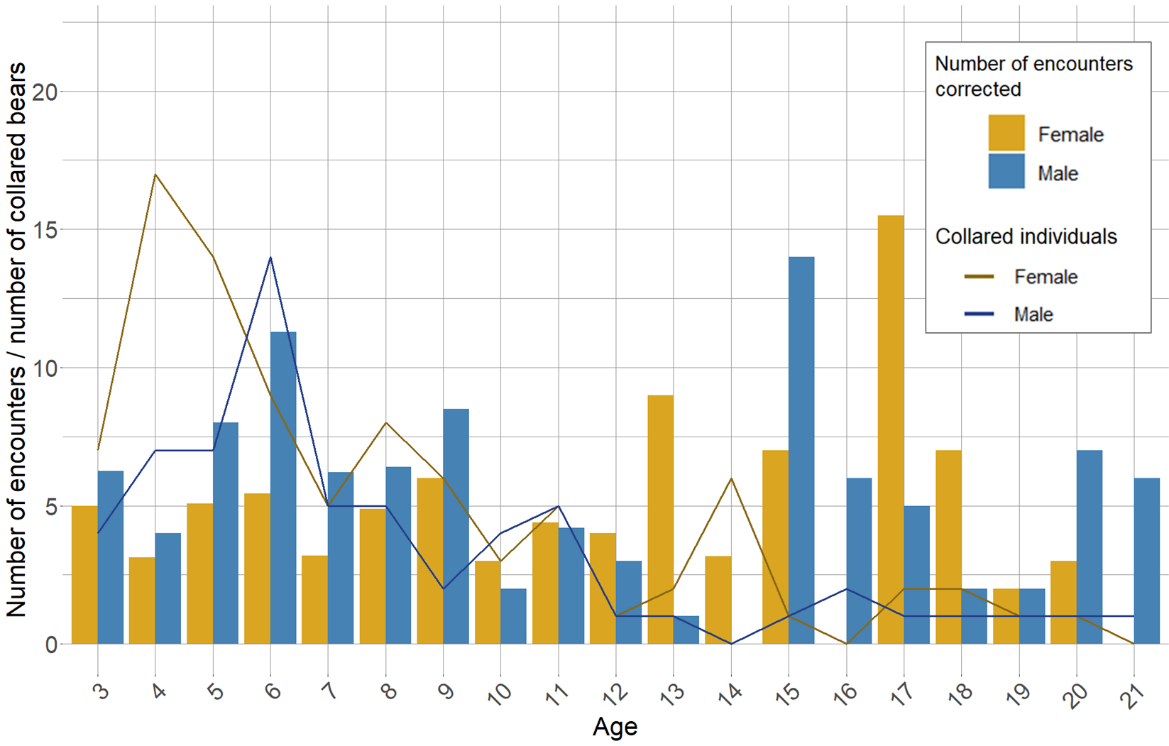


**Table S1.** Descriptive statistics of model terms included in the models to assess habitat selection of female and male brown bears while solitary and when in consorting paths during the mating season in south-central Sweden (2006-2016). Land cover categories are expressed in percentages as they represent absence or presence (0-1).


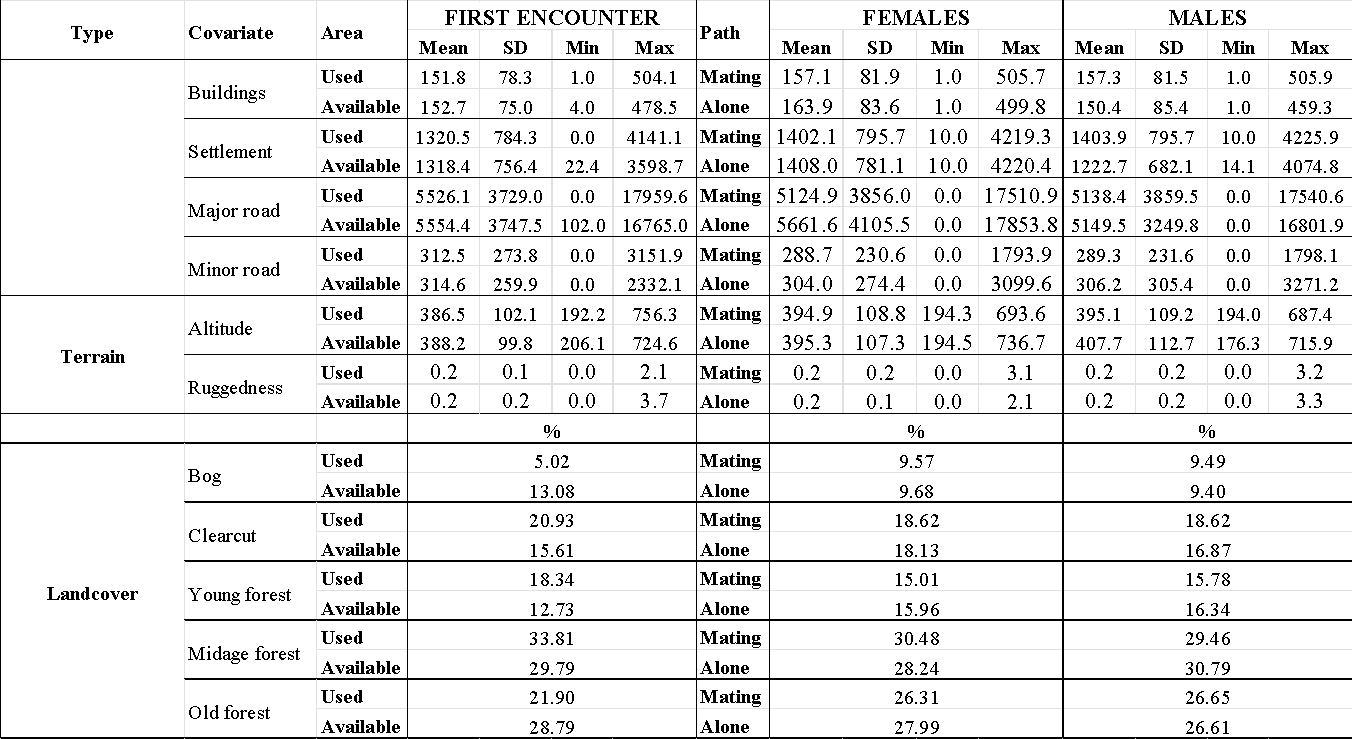


**Table S2.** Formula and results of the model evaluating habitat selection of brown bears in their first consorting encounter compared to the surrounding area (1745m radius buffer) during the mating season in south-central Sweden (2006-2016). Habitat type (bog, clearcut, old forest) was a factor and Mid-aged forest was absorbed into the intercept (reference category). The factor bear is 0 for available habitat and 1 for habitat around the first encounter location.

*mfirst <- glmer(bear ~ as.factor(Habitat) + Buildings + Settlement + MajorRoads + MinorRoads + Elevation + Ruggedness + (1|enctid) + (1|male) + (1|female), data=first, family="binomial", na.action="na.fail", control = glmerControl(calc.derivs = FALSE))*

|  | **Estimate** | **Std. Error** | **z value** | **Pr(>\|z\|)** |
| --- | --- | --- | --- | --- |
| Intercept | -1.275 | 0.142 | -8.980 | < 2e-16 *** |
| Bog | -1.069 | 0.054 | -19.970 | < 2e-16 *** |
| Clearcut | 0.224 | 0.038 | 5.956 | 2.59e-09 *** |
| Old forest | -0.482 | 0.036 | -13.555 | < 2e-16 *** |
| Young forest | 0.287 | 0.040 | 7.146 | 8.93e-13 *** |
| Buildings | 0.138 | 0.038 | 3.682 | 2.31e-04 *** |
| Settlement | 0.170 | 0.063 | 2.715 | 6.623e-03 ** |
| Major Roads | 0.155 | 0.066 | 2.357 | 0.018 * |
| Minor Roads | 0.062 | 0.024 | 2.538 | 0.011 * |
| Elevation | 0.078 | 0.120 | 0.654 | 0.513 |
| Ruggedness | 0.215 | 0.021 | 10.427 | < 2e-16 *** |

**Table S3.** Formula and results of the model performed to evaluate habitat use of female and male brown bears while solitary compared to when in consorting paths during the mating season in south-central Sweden (2006-2016). The factor bear is 0 for solitary and 1 for consorting paths.

*mpaths <- glmer(bear ~ sex*(as.factor(Habitat) + Buildings + Settlement + MajorRoads + MinorRoads + Eelevation + Ruggedness) + Length + (1|pathid) + (1|idindividual), data=path, family="binomial", na.action="na.fail", control = glmerControl(calc.derivs = FALSE))*

|  | **Estimate** | **Std. Error** | **z value** | **Pr(>\|z\|)** |
| --- | --- | --- | --- | --- |
| Intercept | 0.568 | 0.0846391 | 6.707 | 1.98E-11*** |
| Sex M | -0.176 | 0.1171047 | -1.505 | 0.132 |
| Bog | -0.001 | 0.034361 | -0.014 | 0.989 |
| Clearcut | -0.062 | 0.0272843 | -2.261 | 0.02375* |
| Old forest | -0.111 | 0.0246037 | -4.514 | 6.35e-06** |
| Young forest | -0.166 | 0.0288754 | -5.735 | 9.74e-09*** |
| Buildings | 0.034 | 0.0210587 | 1.618 | 0.106 |
| Settlement | -0.081 | 0.0261983 | -3.082 | 0.002** |
| Major Roads | -0.679 | 0.0275702 | -24.641 | < 2e-16 *** |
| Minor Roads | -0.106 | 1.51E-02 | -7.019 | 2.24e-12*** |
| Elevation | 0.074 | 0.0671303 | 1.097 | 0.27258 |
| Ruggedness | 0.164 | 0.0156706 | 10.464 | < 2e-16 *** |
| Sex M: Bog | 0.043 | 0.0487275 | 0.884 | 0.378 |
| Sex M: Clearcut | 0.223 | 0.0387477 | 5.745 | 9.17e-09*** |
| Sex M: Old forest | 0.171 | 0.0347531 | 4.916 | 8.85e-07*** |
| Sex M: Young forest | 0.213 | 0.0405367 | 5.261 | 1.43e-07*** |
| Sex M: Buildings | 0.539 | 0.0299315 | 18.02 | < 2e-16 *** |
| Sex M: Settlement | 0.431 | 3.53E-02 | 12.233 | < 2e-16 *** |
| Sex M: Major Roads | 0.441 | 3.53E-02 | 12.483 | < 2e-16 *** |
| Sex M: Minor Roads | 0.005 | 2.09E-02 | 0.261 | 0.794 |
| Sex M: DEM | -1.017 | 0.0856025 | -11.885 | < 2e-16 *** |
| Sex M: Ruggedness | -0.148 | 2.17E-02 | -6.817 | 9.28e-12*** |
| Length | -0.089 | 2.89E-02 | -3.082 | 0.002 ** |
